# P06 The effect of ciprofloxacin prophylaxis during haematopoietic cell transplantation on infection episodes, exposure to treatment antimicrobials and antimicrobial resistance: a single-centre retrospective cohort study

**DOI:** 10.1093/jacamr/dlad143.010

**Published:** 2024-01-03

**Authors:** Ioannis Baltas, Konstantinos Kavallieros, Giannis Konstantinou, Eirini Koutoumanou, Malick M Gibani, Mark Gilchrist, Frances Davies, Jiri Pavlu

**Affiliations:** Department of Infection, Immunity and Inflammation, Institute of Child Health, University College London, London, UK; Department of Haematology, Hammersmith Hospital, Imperial College Healthcare NHS Trust, London, UK; Faculty of Medicine, Imperial College London, London, UK; Faculty of Medicine, Imperial College London, London, UK; Population, Policy & Practice Research and Teaching Department, Great Ormond Street Institute of Child Health, University College London, London, UK; Department of Infectious Disease, Faculty of Medicine, Imperial College London, London, UK; Department of Infectious Disease, Faculty of Medicine, Imperial College London, London, UK; Department of Infectious Disease, Faculty of Medicine, Imperial College London, London, UK; Department of Infectious Disease, Imperial College NHS Healthcare Trust, St Mary's Hospital, London, UK; Department of Haematology, Hammersmith Hospital, Imperial College Healthcare NHS Trust, London, UK; Faculty of Medicine, Imperial College London, London, UK

## Abstract

**Background:**

Fluroquinolone prophylaxis during haematopoietic cell transplantation (HCT) remains contentious.

**Objectives:**

To determine the effectiveness of ciprofloxacin prophylaxis across different HCT indications and its association with exposure to treatment antimicrobials and antimicrobial resistance.

**Methods:**

All admission episodes for HCT (*N*=400) in a tertiary centre between January 2020 and December 2022 were studied. Allogeneic haematopoietic cell transplantation (allo-HCT) recipients received prophylaxis with ciprofloxacin during chemotherapy-induced neutropenia, while autologous haematopoietic cell transplantation (auto-HCT) recipients did not.

**Results:**

Allo-HCT was performed for 43.3% (173/400) of patients, auto-HCT for 56.7% (227/400). Allo-HCT was associated with an average of 1.01 fewer infection episodes per 100 admission days (95% CI 0.62–1.40, *P*<0.001) compared with auto-HCT. In allo-HCT, the total exposure to all antimicrobials was higher (+24.8 days of therapy [DOT]/100 admission days, *P*<0.001), as was exposure to ciprofloxacin (+40.5 DOT/100 admission days, *P*<0.001). In contrast, exposure to meropenem (−4.5 DOT/100 admission days, *P*=0.02), piperacillin/tazobactam (−5.2 DOT/100 admission days, *P*<0.001), aminoglycosides (−4.5 DOT/100 admission days, *P*<0.001), glycopeptides (−6.4 DOT/100 admission days, *P*<0.001), was reduced. Enterobacteriaceae isolated during allo-HCT were more resistant to ciprofloxacin (65.5%, 19/29 versus 6.1%, 2/33, *P*<0.001), ceftriaxone (65.5%, 19/29 versus 9.1%, 3/33, *P*<0.001), and other antimicrobial classes. Vancomycin-resistant enterococci were more common in allo-HCT recipients (11%, 19/173 versus 0.9%, 2/227, *P*<0.001). Inpatient mortality during allo- and auto-HCT was 9.8% (17/173) and 0.4% (1/227) respectively (*P*<0.001). An invasive bacterial infection was documented in 31.3% (125/400) of cases. Allo-HCT patients were more likely to have an invasive Gram-positive bacterial infection (23.1% versus 12.8%, *P*=0.01), while a difference was not observed for invasive Gram-negative bacterial infections (19.7% versus 18.5%, *P*=0.77). Among auto-HSCT recipients, patients with germ cell tumours had the highest probability (*P* for trend 0.03) of recording an invasive bacterial infection (12/23, 52.2%, 95% CI 31.3–74) compared with other indication for auto-HCT.

**Conclusions:**

Ciprofloxacin prophylaxis in allo-HCT was associated with fewer infection episodes and reduced exposure to treatment antimicrobials. Mortality in auto-HCT remained low. A significant burden of antimicrobial resistance was detected in allo-HCT recipients. HCT recipients due to germ cell tumours, not receiving ciprofloxacin prophylaxis, recorded the highest incidence of invasive bacterial infections and represent a possible target group for this intervention.

